# Evaluation of Eleview^®^ Bioadhesive Properties and Cushion-Forming Ability

**DOI:** 10.3390/polym12020346

**Published:** 2020-02-05

**Authors:** Valentina Giannino, Lucia Salandin, Cristina Macelloni, Luigi Maria Longo

**Affiliations:** 1 Cosmo SpA; Via C. Colombo 1, 20020 Lainate (MI), Italy; vgiannino@cosmopharma.com (V.G.); lsalandin@cosmopharma.com (L.S.); 2 Cosmo Pharmaceuticals NV, Riverside II, Sir Rogerson’s Quay, D02 KV60 Dublin, Ireland

**Keywords:** Eleview®, endoscopic-mucosal resection (EMR), endoscopic submucosal dissection (ESD), bioadhesion, cushion forming ability

## Abstract

Submucosal injection is generally required for both endoscopic-mucosal resection (EMR) and submucosal dissection (ESD). SIC-8000 (Eleview®) is a new liquid composition in the form of a microemulsion for submucosal injection, approved by the Food and Drug Administration (FDA) 510(k) and Conformité Européene (CE) marked, containing a biocompatible polymer as a cushioning agent. The aim of this study was to evaluate Eleview®’s performance in terms of bioadhesive properties and cushion-forming ability. The bioadhesion was evaluated by measuring the interaction between Eleview® and the extracellular matrix (the main component of the submucosal layer) using the texture analyzer. To better comprehend the mechanism of action of Eleview® after submucosal injection, force of detachment and adhesion work were measured for the following formulations: Eleview®, Eleview® without poloxamer (functional polymer), poloxamer solution alone, normal saline, and MucoUp® (competing product on the Japanese market). The results obtained show the interaction between Eleview® and the extracellular matrix, highlighting the stronger bioadhesive properties of Eleview® compared with Eleview® without poloxamer, poloxamer solution alone, as well as normal saline and MucoUp®. The ability of Eleview® to form a consistent and long-lasting cushion in situ, once injected into the submucosal layer, was tested ex vivo on a porcine stomach. The results obtained show a better permanence in situ for the product compared with normal saline injection and to MucoUp® (*t*-test, *p* < 0.05).

## 1. Introduction

Endoscopic mucosal resection (EMR) and endoscopic submucosal dissection (ESD) are being increasingly used worldwide for endoscopic ablation of esophageal and gastric cancers, as well as colon polyps. These techniques are preferred over invasive surgery given their favorable outcomes. Research is underway to make these endoscopic procedures safer, simplified, and time-efficient, including the development of new tools for dissection, hemostasis, and submucosal elevation prior to resection. 

An important aspect of successful endoscopic resection, particularly for non-polypoid and flat mucosal polyps, is a high and long-lasting elevation through a submucosal cushion, in which a biocompatible fluid is injected between the lesion and deeper submucosal layers. Submucosal injection allows a safe and complete removal of the target lesions and decreases the risk of adverse events such as bleeding and perforation [1].

The cushion lifts the lesion, facilitating its capture and removal in EMR, with the aid of a snare; in ESD, the cushion minimizes mechanical or electrocautery damage to the deeper layers of the gastrointestinal (GI) wall [2]. In ESD, agents for submucosal lifting are first injected around the perimeter of the lesion to provide a margin of safety when excising the mucosa [3].

Until recently, the substances used for injection during endoscopic resection procedures were limited to normal saline or other volume-expanding agents designed to be administered intravenously and were only subsequently adopted for use during endoscopy. All the solutions used in the past for submucosal injection were not approved for such indication and were used off-label. A blue dye (usually methylene blue or indigo carmine) was generally added to the lifting agent to provide a clearer visualization of the lesion borders [4].

Eleview^®^ is an FDA 510(k) cleared class II and CE marked medical device [5]. The component ingredients of Eleview^®^ include water for injection, medium-chain triglycerides, poloxamer 188, polyoxyl-15-hydroxystearate, sodium chloride, and methylene blue [6]. Eleview^®^ is a ready-to-use injectable liquid microemulsion composition intended for use in gastrointestinal endoscopic procedures for the submucosal lifting of polyps, adenomas, early stage tumors, or other gastrointestinal mucosal lesions, before their removal with a snare or other endoscopic device.

A recently published, randomized, double-blind clinical study compared the use of Eleview^®^ as a submucosal injectate with normal saline solution (added with methylene blue) in EMR for large (≥2 cm) colorectal polyps. Eleview^®^ showed a statistically significant improvement (*p* < 0.001) in total volume needed for resection, volume per lesion size, initial injected volume, and Sidney Resection Quotient compared to the reference comparator. Additionally, the study showed positive trends for lower time of resection, lower number of resection pieces, and higher en bloc resection rate in the Eleview^®^ arm compared with normal saline. No statistical difference was found on adverse events between the two arms (SIC-8000, 18.6%; saline solution, 17%; *p*-value: 0.86) [7,8]. 

Eleview^®^ is available on the market in strips of five 10 mL plastic ampoules provided with an easy twist-off cap. Eleview^®^ is drawn from the ampoule into a 10 mL luer lock syringe and injected with a standard injection needle. Once injected into the submucosa beneath the lesion, Eleview^®^ forms an immediate submucosal cushion of optimal height, consistency, and duration, which remains localized in the injection area with limited lateral diffusion, enabling an easy and safe resection with the common endoscopic resection tools. In addition, Eleview^®^ provides a long-lasting submucosal cushion that has been shown to last up to 45 min or more, regardless of mucosal cuts [9,10,11,12,13].

The aim of the present study was to evaluate the bioadhesive properties of Eleview^®^, to assess whether a certain interaction occurs between Eleview^®^ and the extracellular matrix (the main component of submucosa), and to measure its ability to form a cushion and its duration.

The extracellular matrix (ECM) used in the present study was a non-cellular three-dimensional macromolecular network derived from a porcine source (composed of porcine small intestine submucosa (SIS)), which was selected to mimic the submucosa.

To better comprehend Eleview^®^’s action mechanism after submucosal injection, force of detachment (Fmax; mN) and adhesion work (AUC; mN·mm) were measured on the following test formulations: Eleview^®^, Eleview^®^ without poloxamer 188, an aqueous solution of poloxamer 188 at the same concentration of Eleview^®^, normal saline, and MucoUp^®^.

The cushion-forming ability performed on ex vivo porcine stomach to evaluate Eleview^®^’s ability to form a consistent and long-lasting cushion in situ, once injected into the submucosal layer. The same test was performed with MucoUp^®^ and normal saline.

## 2. Materials and Methods

### 2.1. Materials

- Eleview^®^, Cosmo Technologies Ltd, Dublin, Ireland
- Exrtacellular Matrix: Oasis Wound Matrix, Smith & Nephew, Inc., Fort Worth, TX, USA
- Eleview^®^ without poloxamer 188. This formulation was prepared at Pharmaceutical Development department of Cosmo SpA, Lainate (MI), Italy
- Poloxamer 10% solution. This solution was prepared at Pharmaceutical Development department of Cosmo SpA, Lainate (MI) Italy. Poloxamer 188 was provided by BASF, Geismar, LA, USA
- Normal saline (0.9% NaCl), Eurospital, Trieste, Italy
- Frozen cleaned porcine stomach, Area Qualità S.r.l., Borghetto di Borbera (AL), Italy
- MucoUp^®^, Seikagaku Corporation, Tokyo, Japan

Poloxamers 188 is a non-ionic poly (ethylene oxide) (PEO)–poly (propylene oxide) (PPO) copolymer with molecular weight ranging from 7680 to 9510 Da. It shows inverse thermosensitivity; therefore, it is soluble in aqueous solutions at low temperatures, but will gel at higher temperatures [14].

MucoUp^®^ was selected for comparison since it is a commercial product on the Japanese market with the same intended use as Eleview^®^. MucoUp^®^ is only available in Japan and not in Europe or in the United States. The component ingredients of MucoUp^®^ include water for injection, purified sodium hyaluronate, sodium chloride as a tonicity agent, NaH_2_PO_4_, and Na_2_HPO_4_·12H_2_O.

Normal saline was tested since it is the most commonly used submucosal injection agent during endoscopic resection procedures (standard of care).

The porcine stomach was selected as a testing system since it is a widely accepted model of the human gastrointestinal mucosa [15,16,17,18,19,20]. The scientific literature on submucosal injection agents describes the use of this model to evaluate the performance of the different agents in terms of height and duration of the submucosal cushion [21,22,23,24,25,26,27,28]. 

### 2.2. Bioadhesive Test

**Figure 1:**
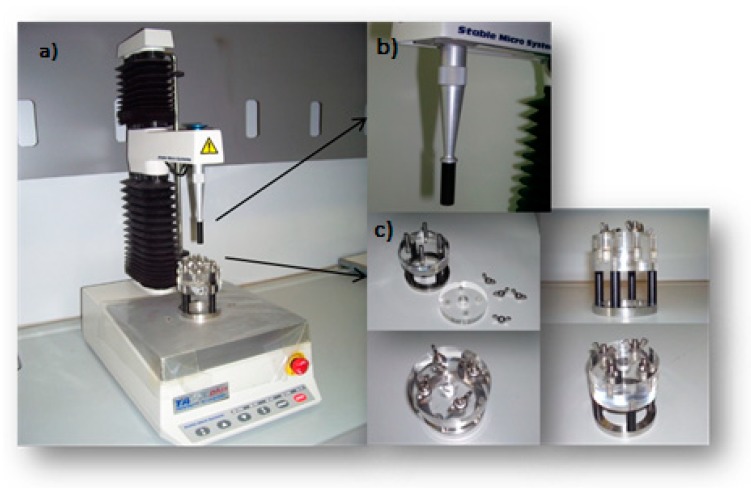
Scheme of the tensile tester. The texture analyzer (**a**) consisted of a probe (**b**) and a support (**c**).

Bioadhesive properties of all the test items were assessed at 37 °C using means of a TA-XT Plus Texture analyzer (ENCO, Spinea, I; Figure 1a) equipped with 1 kg load cell and Mucoadhesion Rig (A/MUC) measuring system.

The TA-XT Plus Texture analyzer consists of a probe (Figure 1b) (Ø: 10 mm) composed of Teflon and a support (Figure 1c) composed of plexiglass. The support consists of two cylinders: the lower serves as a base for the sample location, the upper has a circular hole in the center (Ø: 14 mm), which enables probe movement inside. A cylindrical probe (P/10, Ø 10 mm, Cyl Delrin) was fixed to the vertical bracket of the instrument.

The cylindrical probe, depending on the test conditions, was fixed with a bi-adhesive tape and an extracellular matrix disc (Ø: 10 mm) or normal saline (blank) layered on a filter paper disc (Ø: 10 mm). Then, an exact volume (40 µL) of sample was layered on a filter paper disc fixed to the support.

**Figure 2:**
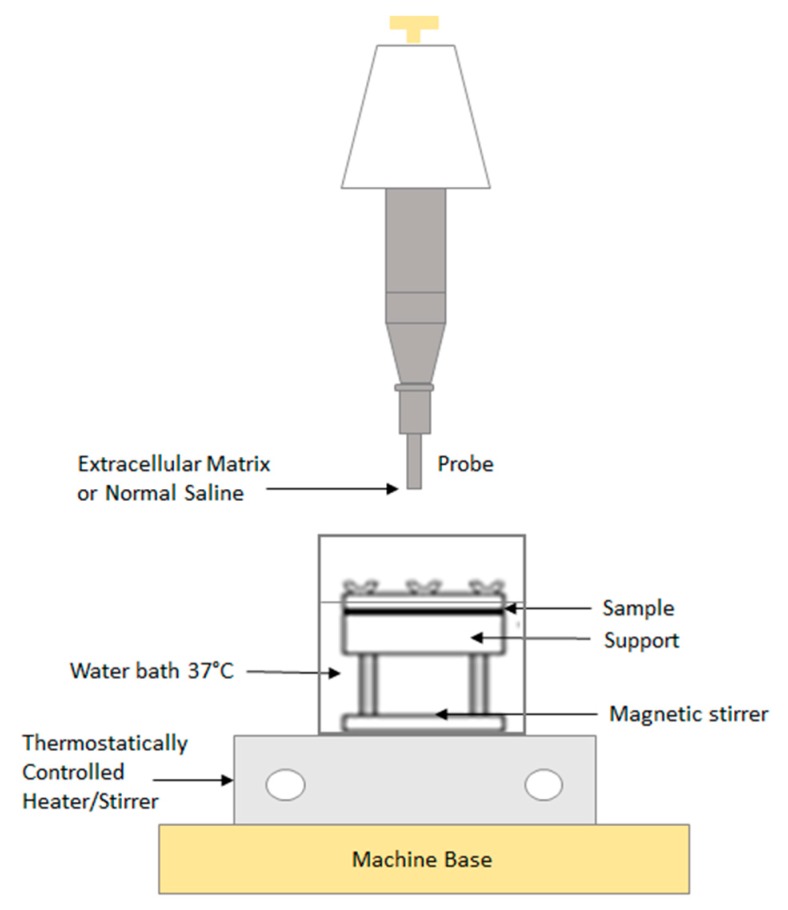
Scheme of bio-adhesive tests set-up.

The cylindrical probe was placed in contact with the sample, maintained at 37 °C, applying a 2500 mN preload for 3 min, and then the probe was lifted at 2.5 mm/min up until the complete separation of the interface [29]. All the bioadhesion measurements were recorded at the Department of Drug Science, University of Pavia, using the above-mentioned parameters. The scheme of the set-up is reported in Figure 2.

The force of detachment as a function of displacement was recorded and the parameters maximum force of detachment (Fmax; mN) and work of adhesion (AUC; mN·mm) were considered. Fmax is the maximum force required to separate the sample from the substrate. AUC is the work needed to overcome the attractive force between the surface of the sample and the surface of another material (for example, the extracellular matrix). The force required to detach the sample from the substrate (Fmax) was measured as a peak value, whereas the work of adhesion (AUC) was calculated as area under the force versus displacement curve.

The following measurements were recorded:

The first set of analyses were performed to evaluate the bioadhesive property of the Eleview^®^ and its components upon contact with the extracellular matrix or with normal saline (blank).

The following items were analyzed:

- Eleview^®^
- Eleview without poloxamer
- Poloxamer solution

The second set of analyses were performed to compare the bioadhesive behavior of Eleview^®^, normal saline, and MucoUp^®^ upon contact with the extracellular matrix.

The following items were analyzed:

- Eleview^®^
- Normal saline
- MucoUp^®^

For all the sets of analyses, Fmax and AUC were measured. Six replicates were considered for each sample.

To assess the bioadhesive behavior of Eleview^®^ compared to the standard of care normal saline, ΔFmax and ΔAUC parameters were calculated as the difference between the values obtained for Eleview^®^, its components (Eleview without poloxamer 188 and poloxamer 188 solution), and normal saline. 

ΔFmax was calculated according to the following equation:

ΔFmax = (Fmax_sample_ − Fmax_saline_)/Fmax_saline_

where Fmax_sample_ was the maximum force measured in presence of the extracellular matrix and Fmax_saline_ was the maximum force measured for the normal saline [30].

### 2.3. Cushion-Forming Ability Test

**Figure 3:**
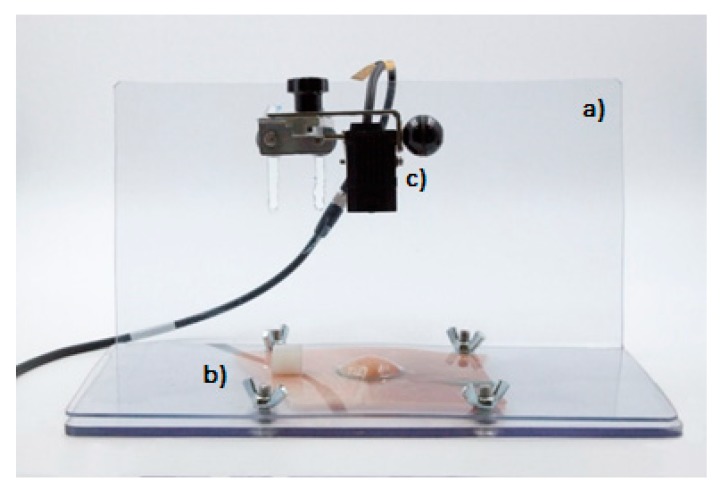
Multifunctional analog laser sensor assembled on the plexiglass support.

The cushion-forming ability of Eleview^®^ was evaluated using a multifunctional analog laser sensor (Mod IL-065) equipped with Datasink 100 software, which is able to elaborate the results in terms of cushion height decreasing (mm) as a function of time (Figure 3).

The equipment, materials, and methods used for the test are described below:

- Plexiglass support base (dimensions: b 27 × 15 cm^2^; height 15 cm) (Figure 3a)
- Mobile perforated plexiglass part (dimensions: 27 × 15 cm^2^; hole Ø 3 cm) (Figure 3b)
- Multifunctional analog laser sensor Mod IL-065 (Figure 3c). The instrument was used to detect cushion height variations (mm) through a multifunctional analog laser sensor (Mod IL-065) equipped with a graphic interface (software: Datasink100), which allowed to capture the cushion height every minute, then to record and to elaborate the data collected.

The cushion-forming ability test was performed using a 10 × 10 cm^2^ portion of porcine stomach cut and cleaned after defrosting and maintained at 37 °C.

The procedure for obtaining porcine stomach is reported below:

- Thaw the frozen porcine stomach and keep it at 37 °C in a thermal blanket;
- Remove the packaging, open the stomach using a bistoury, and clean the mucosa using disposable paper towels;
- Cut a 10 × 10 cm^2^ portion of the stomach using a scalpel and place it on the plexiglass base; and
- Fix the stomach portion with the mobile perforate plexiglass part (hole diameter: 3 cm), tightening the appropriate screws.

After resetting the laser sensor, 5 mL of Eleview^®^, normal saline, or MucoUp^®^ were slowly injected from a 10 mL disposable luer lock syringe with a needle into the sub-mucosal layer to form a cushion. The cushion height over time was recorded after product injection every minute, for a minimum of 45 min, with the laser sensor. Six replicates were considered for each sample.

The results are expressed as cushion height decrease, which was calculated by the following equation:

Cushion height decrease (%) = ((Height_0_ − Height_45_)/Height_0_) × 100

where Height_0_ and Height_45_ are the cushion height at t_0_ = 0 and after 45 minutes, respectively.

### 2.4. Statistical Analysis

Experimental values were subjected to statistical analysis carried out using the statistical package Statgraphics 5.0 (Statistical Graphics Corporation, Rockville, MD, USA). In particular, a one-way ANOVA multiple range test was used.

## 3. Results and Discussion

### 3.1. Bioadhesive Test

#### 3.1.1. Adhesion Evaluation of Eleview^®^, Eleview^®^ without Poloxamer, and Poloxamer

The bioadhesive behavior of Eleview^®^ and its components were studied, evaluating their intrinsic adhesive properties in comparison with their interaction with a biological substrate, ECM.

Therefore, Eleview^®^, Eleview^®^ without poloxamer 188, and poloxamer 188 solution were tested both after interaction with a non-biological substrate, used as the blank (normal saline), and with a biological substrate, the ECM.

**Figure 4:**
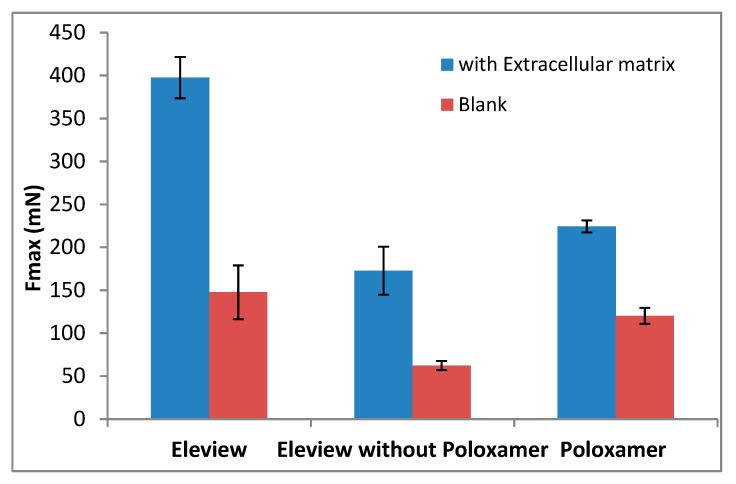
Force of detachment values with or without extracellular matrix (mean values ± standard error (SE); *n* = 6).

**Figure 5:**
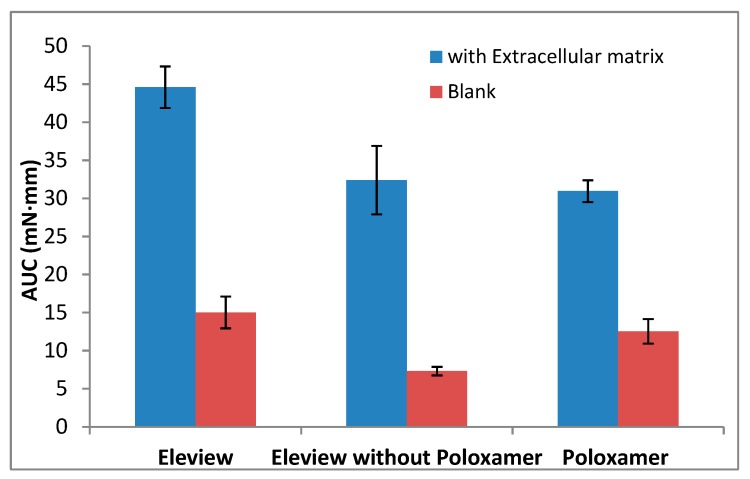
Adhesion work values obtained with or without extracellular matrix (mean values ± SE; *n* = 6).

The results of Fmax (mN) and AUC (mN·mm) are shown respectively in Figure 4 and Figure 5.

The results demonstrated that bioadhesion is significantly driven by interaction with ECM. The low results obtained for Fmax and AUC parameters without ECM confirm the weak adhesive properties of all the samples tested in absence of the biological substrate, ECM. A significant increase (one-way ANOVA, multiple range test, p < 0.05) in Fmax and AUC values (Figure 4 and Figure 5) was observed in the presence of extracellular matrix in comparison with the blank. 

The data obtained without ECM (with non-biological substrate, normal saline) showed that Fmax values (red data in Figure 4) of Eleview^®^ were higher than the values obtained for Eleview^®^ with and without poloxamer and for poloxamer solution, even if the difference was only statistically significant with respect to Eleview^®^ without poloxamer (*t*-test, p < 0.05), meaning that the poloxamer solution has intrinsic bioadhesive properties confirmed by the statistical difference (*t*-test, p < 0.05) with the formulation without the polymer. 

The AUC values obtained with the non-biological substrate, Blank (red data in Figure 5), confirm the trend (i.e., a statistically significant difference between Eleview^®^ and Eleview^®^ without poloxamer (*t*-test, p < 0.05) was found).

Upon contact with ECM, the trend (Eleview^®^ > poloxamer solution > Eleview without poloxamer) was the same observed for the blank measurement, but a significant increase was observed for all the samples for both Fmax and AUC parameters, which are indexes of bioadhesive properties.

The significantly higher values (one-way ANOVA, multiple range test, p < 0.05) of Fmax and AUC parameters obtained for Eleview^®^ compared with the other two samples (Eleview^®^ without poloxamer and poloxamer solution) indicated that the entire formulation, rather than the individual components, generates a significant increase in Fmax and AUC. We found no statistically significant difference (*t*-test) between poloxamer solution and Eleview^®^ without poloxamer for both Fmax and AUC results, even if Poloxamer solution shows higher bioadhesive properties than Eleview^®^ without Poloxamer.

This result confirms that Eleview^®^ interacts considerably with the extracellular matrix and, due to this interaction, it has bioadhesive properties. Eleview^®^‘s interaction with the glycoproteins of the ECM is governed by nonspecific and non-covalent bonds (electrostatic, hydrogen, and hydrophobic), mainly driven by poloxamer, which can act as an H-bond acceptor due to the presence of hydrophilic oxide groups in its chain [31].

#### 3.1.2. Bioadhesion Evaluation of Eleview^®^ vs. Normal Saline and MucoUp^®^

The bioadhesive behavior of Eleview^®^ and its components was compared with two compounds largely used as submucosal injection agents in endoscopy today (normal saline and MucoUp^®^) upon contact with extracellular matrix to mimic in vivo clinical conditions. 

**Figure 6:**
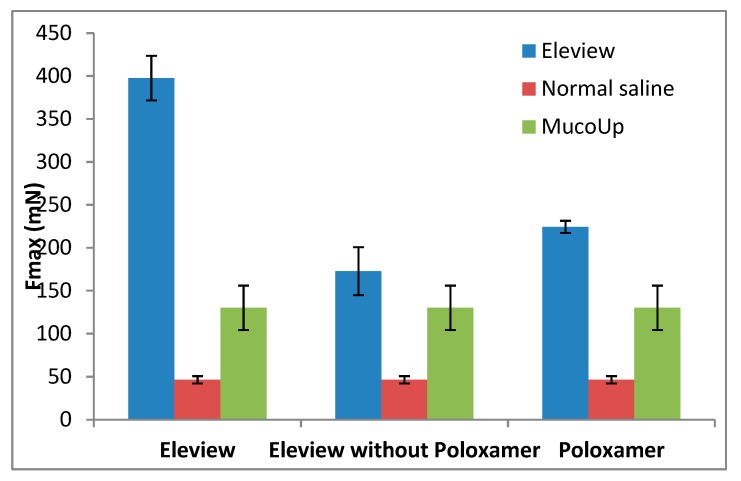
Force of detachment values from extracellular matrix: Eleview^®^ vs. normal saline and MucoUp^®^ (mean values ± SE; *n* = 6).

**Figure 7:**
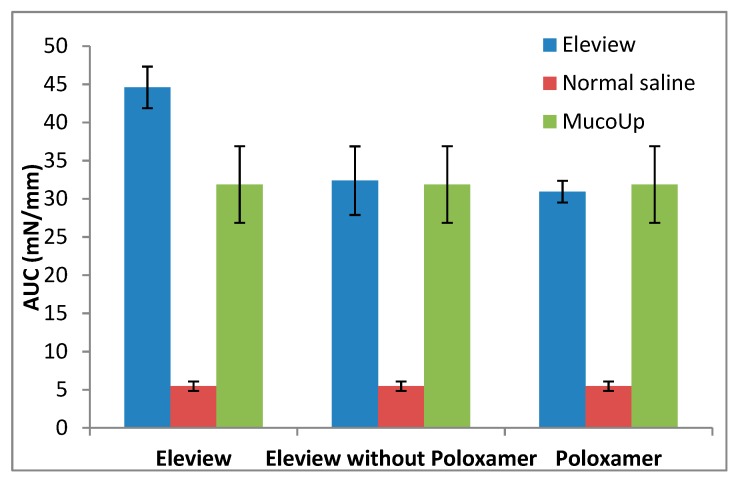
Adhesion work values from extracellular matrix: Eleview^®^ vs. normal saline and MucoUp^®^ (mean values ± SE; *n* = 6).

Fmax (mN) and AUC (mN·mm) results are respectively shown in Figure 6 and Figure 7.

In the present study, the bioadhesive interaction of Eleview^®^ with ECM, as shown in Figure 6 and Figure 7, was higher compared with normal saline and MucoUp^®^, supporting the better performance of Eleview^®^ compared to the two comparators.

Eleview^®^, its components, and MucoUp^®^ have statistical superiority (one-way ANOVA, multiple range test, p < 0.05) compared with normal saline. 

The results confirmed that a solution of an inorganic salt like normal saline, widely used in endoscopic procedures to lift the submucosal layer, shows a weak interaction with the structure of the ECM compared with formulations containing polymers with a number of hydrophilic groups, which are able to actively coordinate and interact with the surrounding water molecules.

The findings demonstrated that Eleview^®^, as well as poloxamer solution, are characterized by higher bioadhesive properties than MucoUp^®^ and result in statistically superior Fmax values for Eleview^®^ and Poloxamer solution compared to MucoUp^®^ (one-way ANOVA, multiple range test, p < 0.05). On the contrary, with regards to AUC, we observed a trend in the superiority of Eleview^®^ versus MucoUp^®^, although the difference did not reach statistical significance.

These results suggest that the Eleview^®^ formulation is characterized by strong bioadhesive properties, probably due to the interaction of the components of Eleview^®^ (microemulsion and poloxamer together) with the structure of the ECM. This physical interaction allows Eleview^®^ to structure itself in contact with submucosa, probably due to the presence of both poloxamer chains and the other components of the formulation, which act to improve the adhesion properties of Eleview^®^. This means that Eleview^®^, in contact with the submucosa, develops an efficient interaction able to allow it to structure itself. We also confirmed that the entire formulation, rather than the individual components, is responsible for the generation of the significant increase in the Fmax and AUC.

**Figure 8:**
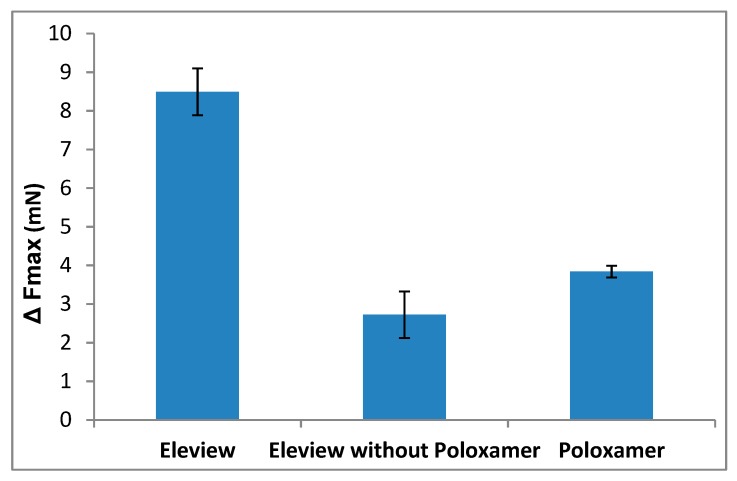
ΔForce of detachment values: difference between the samples and normal saline (mean values ± SE; *n* = 6).

**Figure 9:**
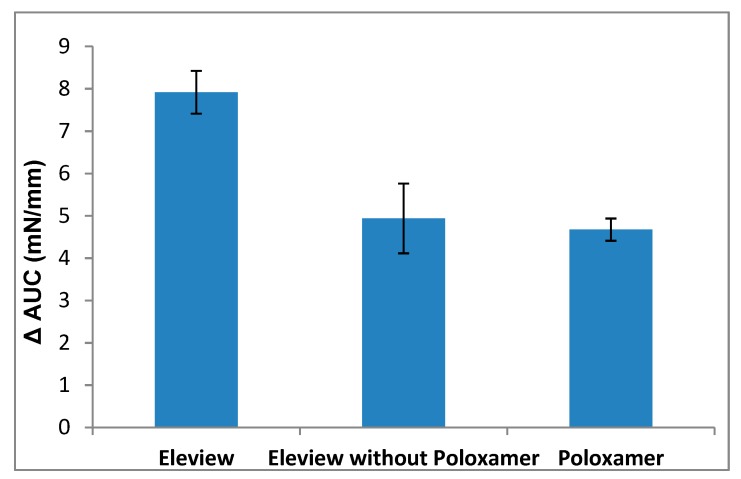
ΔAUC values: difference between the samples and normal saline (mean values ± SE; *n* = 6).

To underline the bioadhesive superiority of Eleview^®^ compared to normal saline, ΔFmax and ΔAUC parameters were calculated as the difference between the values obtained for Eleview^®^, Eleview^®^ without poloxamer, poloxamer 188 solution, and normal saline. The results are shown in Figure 8 and Figure 9. 

Eleview^®^ is characterized by a normalized ΔFmax value significantly higher than that of the other two samples (*t*-test, p < 0.05), indicating a better capability to adhere to the ECM at physiological temperature. The lower ΔFmax values of the other two samples indicate weaker bioadhesive properties than Eleview^®^. 

The ΔFmax values obtained for Eleview^®^ without poloxamer and for the poloxamer solution were not statistically different (*t*-test, p < 0.05). The same statistical results were obtained for ΔAUC values (*t*-test, p < 0.05).

### 3.2. Cushion-Forming Ability

**Figure 10:**
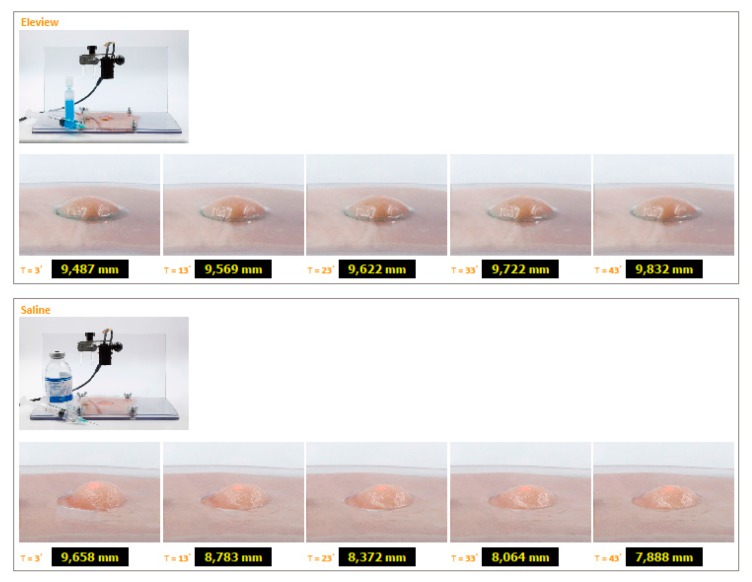
Cushions generated after submucosal injection of Eleview^®^ and normal saline into the submucosa of porcine stomach and relevant height variations as function of time. Photos were taken to allow the stabilization of the system at the third minute after injection and then in intervals of 10 min.

**Figure 11:**
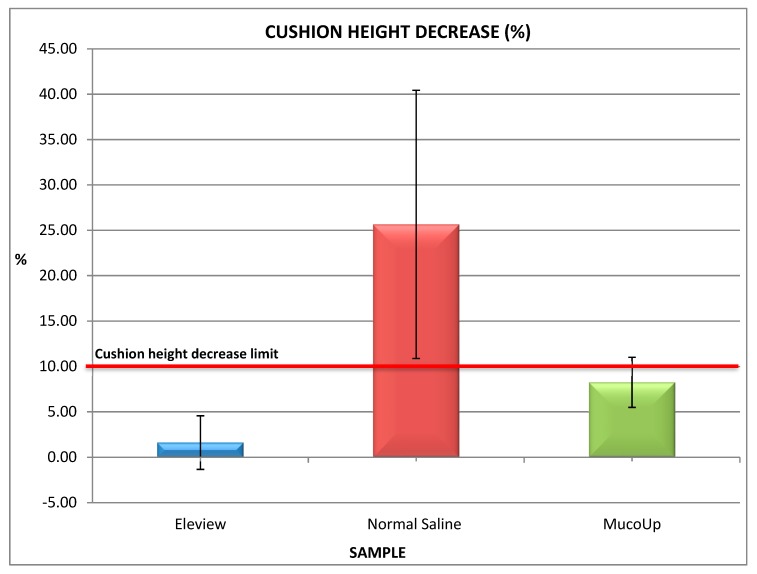
Cushion height decrease values (%) of Eleview^®^ samples, normal saline, and MucoUp^®^ at 45 min (mean values ± standard deviation (SD); *n* = 6).

The results obtained on ex vivo porcine gastric mucosa are shown in Figure 10 and Figure 11.

The findings demonstrated that the duration of mucosa elevation is significantly lengthened by the use of Eleview^®^ compared with normal saline and MucoUp^®^.The results obtained on ex vivo porcine gastric mucosa confirm the bioadhesive data and demonstrate that Eleview^®^ cushion shows a better and longer permanence in situ compared with normal saline or MucoUp^®^ injections (*t*-test, p < 0.05). Eleview^®^ produces less of a cushion height decrease than normal saline as function of time; MucoUp^®^ shows a shorter duration of mucosal elevation than Eleview^®^. Eleview^®^ and MucoUp^®^ also show less variations than normal saline. 

Based on experience, the cushion height decrease limit was fixed to 10%. The values obtained for normal saline were all above this limit; all Eleview^®^’s measurements were under 10% in terms of cushion height decrease. The average obtained for MucoUp^®^ was closer to the limit than Eleview^®^ but under 10%. The poor ability of normal saline to form a long-lasting cushion was also reported in literature as well as the improved mucosa elevation using hyaluronic acid [28], and salt is the functional component of MucoUp^®^.

The mechanism of action of Eleview^®^ upon injection into the area surrounding targeted lesions consists of Eleview^®^’s reconfiguration to create an artificial net to trap water, elevating the mucosal layer from the submucosa and the muscular layer, creating a cushion able to persist for the time needed to perform the polyp removal procedure. The same mechanism is used by MucoUp®, being essentially composed of sodium hyaluronate, a biopolymer with an extensive quantity of hydrophilic groups in its chains, but the natural source of this biopolymer is also a variability factor that could be considered.

## 4. Conclusions

Our findings demonstrated the better performance of Eleview^®^ compared to normal saline and MucoUp^®^, the leading submucosal injection in Japan, confirmed by both bioadhesive and cushion-forming ability results. 

Eleview^®^ and its components are all characterized by intrinsic adhesive properties upon interaction with a non-biological substrate (normal saline); however, according to the data obtained for Fmax and AUC, a significant increase was observed when in contact with the extracellular matrix in comparison with the blank, demonstrating that bioadhesion was significantly driven by the interaction with ECM. 

The interaction of Eleview^®^ with the glycoproteins of the ECM is governed by nonspecific and non-covalent bonds mainly driven by the poloxamer that can act as an H-bond acceptor due to the presence in its chain of hydrophilic oxide groups [31,32].

The comparison of Eleview^®^ with normal saline and MucoUp^®^ demonstrated the stronger bioadhesive interaction of Eleview^®^, demonstrating better performance compared with the two comparators. In terms of bioadhesive behavior, the statistical superiority of Eleview^®^ was observed for Fmax as well as a trend of superiority in terms of AUC compared with MucoUp^®^.

The data of the cushion-forming ability confirmed and reinforced the bioadhesive properties, demonstrating the correlation between high bioadhesion and long-lasting mucosa elevation. Eleview^®^ produced a minor cushion height decrease over time compared with normal saline and MucoUp^®^; therefore, longer permanence in situ upon injection into the submucosa would facilitate endoscopic resection procedures. The bioadhesion properties of Eleview^®^ are particularly important when the piecemeal technique is used for ablating the mucosal lesions. In this technique, the grafting of the cushion to the mucosal layer operated by the bioadhesion forces ensures the survival of the cushion after the partial removal of the lesion, with no need to re-inject a submucosal lift too frequently.

## 5. Patents

Eleview^®^ is object of international patent applications published as WO2015/075024 and WO2015/075015. A list of the patents granted in the U.S. and EU is available at: http://www.cosmopharma.com/activities/patents.
